# Ultrasound-guided in utero injections allow studies of the development and function of the eye

**DOI:** 10.1002/dvdy.21500

**Published:** 2008-03-20

**Authors:** Claudio Punzo, Constance L. Cepko

**Affiliations:** Harvard Medical School, Department of Genetics and Howard Hughes Medical InstituteBoston, Massachusetts

**Keywords:** ultrasound, eye development, mouse, gain and loss-of-function clones, retina, clonal analysis

## Abstract

Ultrasound-guided in utero injections into the brain of murine embryos has been shown to facilitate gene delivery. We investigated whether these methods would allow gene transfer into ocular structures. Gene transfer using retroviral vectors or electroporation was found to be quite effective. We determined the window of time, as well as compared several strains of mice, that yield a high degree of survival and successful gene transfer. Several retroviral constructs were tested for expression and coexpresssion of two genes in retinal cell types. In addition, a retroviral vector was engineered to give cone photoreceptor-enriched expression, and a retroviral vector was demonstrated to provide RNAi-mediated loss-of-function. These methods enable access to early ocular structures and provide a more rapid method of assessment of gene and promoter function than possible using genetically engineered mice.

## INTRODUCTION

The retina is a well-characterized tissue of the central nervous system (CNS) that has served as a model of CNS development, circuitry, and degeneration (Rodieck, 1998). It has a simple layered structure, surrounded by several support tissues, such as the retinal pigmented epithelium (RPE) and sclera. Much of mammalian ocular development occurs in utero, making early experimental perturbations difficult. Traditional genetic methods partially overcome the lack of access, and the recent applications using recombinases such as Cre or Flp (Branda and Dymecki, 2004), allow more precise gain- and loss-of-function experiments. However, even this more recent approach is often limited due to lethality or the lack of appropriate Cre-driver lines that function in the desired spatiotemporal manner. Additionally, most Cre-driver lines affect large populations of cells, complicating the dissection of cell-autonomous gene function. In *Drosophila*, many questions regarding eye development have been answered using elegant clonal loss-of-function strategies that rely upon mitotic recombination (Xu and Rubin, 1993). Although these methods have been established for mice, they involve time consuming and costly procedures that require embryonic stem cells (Liu et al., 2002) and the generation of new mouse lines (Wang et al., 2007).

Previous investigators had used ultrasound-guided injections to successfully target brain structures (Liu et al., 1998; Gaiano et al., 1999). To overcome the obstacles presented above, we investigated whether methods using ultrasound-guided in utero manipulations were also applicable to ocular structures. Despite their much smaller size, we found that gene transfer into ocular structures was successful. We tested several aspects of the procedure to allow for high efficiency, including timing of injections, mouse strains, and gene transfer by means of electroporation and retroviral vectors.

## RESULTS

### Injection Method

**Fig. 1:**
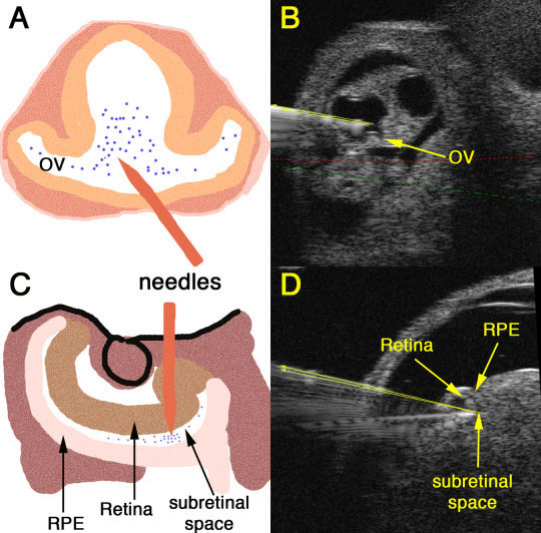
Ultrasound-guided injections. **A**: Schematic representation of developing optic vesicles (OV) at embryonic day (E) 9.5, with the orange arrow indicating the injection needle and blue dots the injected viruses. **B**: Ultrasound image of embryo at E9.5 showing the injection needle (yellow lines) and developing optic vesicles (arrow) and diencephalon. **C**: Schematic representation of developing eye showing retina, retinal pigmented epithelium (RPE), and the subretinal space. The orange arrow indicates the injection needle and blue dots the injected viruses. **D**: Ultrasound image of developing eye at E13.5 showing injection needle (yellow lines), retina, retinal pigmented epithelium, and subretinal space.

Ultrasound-guided in utero injections were performed as early as embryonic day (E) 9.5, to E10.5, and then again between E12.5 and birth. At E9.5, which is before the birth of any retinal cell type, injections were into the lumen of the diencephalon of the developing embryo (Fig. 1A,B). At this time, the lumen of the diencephalon is continuous with that of the optic vesicle, which allows targeting of future retinal and RPE cells. Once the optic cup forms at approximately E10.5, the lumen constricts as the optic stalk forms. Therefore, injections after E10.5 need to be performed directly into the subretinal space (Fig. 1C,D), which is not possible until after E12.5, as the subretinal space is too small to target effectively earlier (Table 1).

**Table 1:** Comparison of Survival and Gene Transfer Efficiency Following Viral Infection and Electroporation^a^

Injection stage	E9.5–E10.5*	E9.5–E10.5†	E9.5–E10.5§	>E12.5
Gene transfer method	Viral infection	Viral infection	Viral infection	Viral infection or electroporation
Location of injection	Diencephalon	Diencephalon	Diencephalon	Subretinal space
Survival rates of mothers	80% 12/15	100% 50/50	96% 62/65	93% 14/15
Survival rates of embryos	67% 103/153	97% 495/510	90% 598/663	80% 130/162
Target efficiency total	47% 31/65	88% 245/278	80% 276/343	71% 59/83
Target efficiency for viral titer 10^6^	43% 15/35	71% 12/17	52% 27/52	71% 22/31
Target efficiency for viral titer 5×10^6^	53% 16/30	75% 18/24	63% 34/54	71% 17/24
Target efficiency for viral titer 10^7^		84% 103/122	84% 103/122	n.d.
Target efficiency for viral titer >10^8^		97% 112/115	97% 112/115	n.d.

a Survival rates of embryos were dependent primarily upon developmental stage and experience with the surgical procedure. They did not depend on litter size, viral vector, or gene transfer method once electroporation conditions were optimized for particular strain of mice. A detailed analysis for CD1 at E9.5-10.5 is as follows:

* values for first 15 surgeries,

† values of remaining surgeries,

§ summary of column * & †;

survival rates of embryos = number of animals born / number of animals expected to be born; target efficiency = number of positive animals / number of infected or electroporated animals. E, embryonic day; n.d., not determined.

Surgeries were performed with isofluorene anesthesia with survival rates >96% for the mother when the surgeries lasted <90 min. Average anesthesia time was 45 min. Survival rates of embryos injected at E9.5–E10.5 was >90%, and targeting efficiency of the eye was >80%. At and after E12.5, survival rates dropped to >80% and targeting efficiency to >70% (Table 1). The decrease in survival at later stages was mainly due to spontaneous abortions of the entire litter. Proper positioning of larger size embryos back into the body cavity of the mother was crucial for prevention of spontaneous abortions. The decrease in target efficiency was mainly due to a reduced targeting area. Three to four embryos were injected per horn, selecting those that were in ideal positions, to minimize manipulation of the embryos. Injections at E9.5–E10.5 usually resulted in both eyes being targeted, because both optic vesicles were open toward the diencephalon. Injections at later stages were performed in only one of the two eyes to reduce the degree of the manipulation. After the surgery, development proceeded with a normal parturition, whereupon pups were suckled and raised by the mother. Strain differences were observed in survival rates of the pregnant mother for the three stains tested, such that CD1 > FVB/N > C57Bl6.

### Gene Transfer Methods

Two methods of gene transfer were tested: retroviral infection and electroporation. These methods have some similarities, as well as some differences, which are relevant for different applications. One similarity is the target cell that takes up the DNA. Viral infection with Type C retroviral vectors as well as electroporation target mitotic progenitor cells. Electroporated plasmid DNA either accesses primarily mitotic cells, as they line the lumen where the DNA is delivered, or there is some selectivity in the type of cells that can successfully translocate the DNA to the nucleus, and/or express detectable levels of reporter genes (Matsuda and Cepko, 2004). Whereas type C retroviruses can integrate only in mitotic cells (Miller et al., 1990), lentiviral vectors can integrate into postmitotic and mitotic cells (Naldini et al., 1996). However, the route of injection into a lumen might only deliver the viral vector to the area adjacent to mitotic cells, so even the lentiviral infections tend to target mitotic cells. Electroporated DNA or viral DNA is then inherited by progeny cells. The aspects that are quite different are the stability of expression and the number of cells targeted, as described further below.

#### Gene transfer by viral vectors

**Fig. 2:**
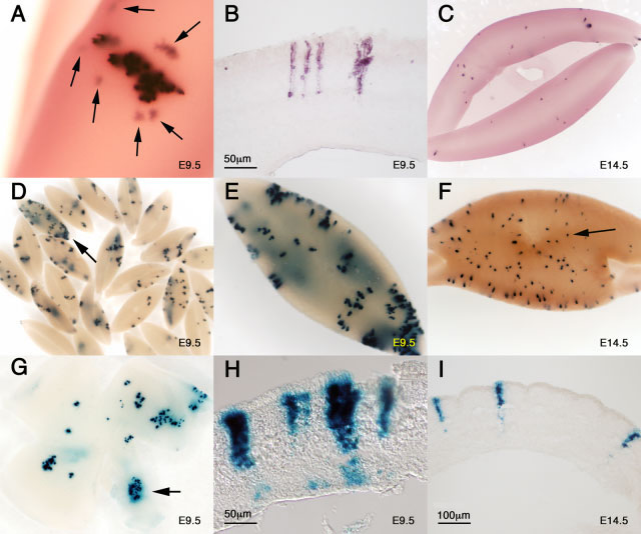
Injections with the pQCXIX vector (pQC-H2BGFP-IX) at embryonic day (E) 9.5 (A–I). **A**–**C**: Cross-section through retina at postnatal day (P) 28 showing nuclear green fluorescent protein (GFP) in all retinal layers. Red shows immunohistochemical signal from anti-red/green cone opsin staining. Non-nuclear green signal shows cone segments labeled with PNA. (A) 4′,6-diamidine-2-phenylidole-dihydrochloride (DAPI); (B) nuclear, GFP encoded by the virus; (C) overlay. **D**: Fluorescence visualized through the lens at P28. **E**: Side view of enucleated eye at P28 with cornea on the left (arrow) and optic nerve to the right. In an albino background, fluorescent retinal, retinal pigmented epithelium (RPE), and scleral clones are seen together. **F**: Brightfield and fluorescence image of an infected retina shown as a flat mount, with photoreceptor surface up at 10 weeks of age showing distribution of retinal clones. **G**,**H**: Flat mount at 10 weeks of age showing different degrees of infection. Viral titer in G was 5 × 10^6^ CFU/ml; in H, 10^7^ CFU/ml (H shows fluorescent signal of F). **I**: Viral titer was 2 × 10^8^ CFU/ml.

**Fig. 3:**
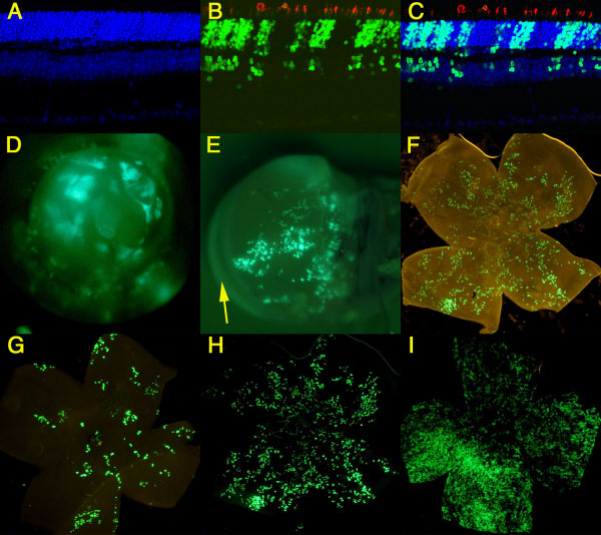
Viral infections with LIA (A–C) and BAG (D–I) showing alkaline phosphatase and X-gal staining, respectively, at postnatal day (P) 14. Injection age is indicated in the lower right of each panel. **A**: Whole-mount view of a single LIA clone that was found to have an expected cellular distribution of ONL and INL cells, as assessed by section analysis, after infection at embryonic day (E) 9.5. Faint horizontal cells (arrows) are visible next to the main columns. **B**: Cross-section through a retina of a LIA clone that did not contain the expected cellular distribution of ONL and INL cells after infection at E9.5. Only photoreceptors in the ONL were seen. **C**: Whole-mount showing LIA clones from E14.5 injections. Size of clones was smaller than those from E9.5 injections (compare H with I). Clones were usually composed of one column (see also F and I). Viral titer was 5 × 10^5^ CFU/ml (compare with F). **D**,**E**: Retinae after BAG infections at E9.5 from 3 surgeries performed in one afternoon. Higher magnification of a retina from D (arrow) is shown in (E). **F**: Retina from an E14.5 injection showing many small clones (an example of a single clone is indicated by the arrow). Viral titer was 5 × 10^6^ CFU/ml (compare with C). **G**: Flat mount of BAG infected retina at E9.5. Arrow points to an example of a single clone that is composed of multiple columns. **H**: Cross-section through a retina infected with BAG at E9.5. All labeled cells are part of a single clone. **I**: Cross-section through retina in (F) showing three individual clones. ONL, outer nuclear layer; INL, inner nuclear layer.

Retroviruses enable indefinite expression, by virtue of their integration into the host genome. Moreover, by controlling the viral titer, one can infect a small number of cells, enabling clonal analysis for lineage studies or distinctions between autonomous and nonautonomous effects. At E9.5–E10.5, a minimal titer of 10^6^ colony forming units/ml (CFU/ml) was required for successful infection, although this only yielded a few clones per eye in successfully infected animals, with a targeting efficiency (percentage of animals injected with retinal clones) of <80%. The targeting efficiency of approximately 80% was achieved with a titer of >10^7^ CFU/ml. If the number of clones desired is small, so that clonal analysis can be conducted, 5 × 10^6^ CFU/ml is an ideal titer as most infected animals will have a few clones per eye. When infecting at E9.5–E10.5, the clones are large; therefore, one needs clones that are well separated to avoid confusion in assignment of clonal boundaries. With titers of >10^8^ CFU/ml, 20–30% of the surface area of the retina had clones (see Fig. 3G–I). For injections into the subretinal space at E12.5 and later, a minimum of 5 × 10^5^ CFU/ml is recommended. Higher concentrations work and increase the number of clones. The clone sizes varied relative to the time of infection, such that earlier infections resulted in larger clones, and later infections resulted in relatively smaller clones (Fig. 2; Turner et al., 1990). As our previous work showed that infections into E13–E14 produced clone sizes that varied tremendously, from 1 to 234 cells with a large standard deviation among >300 clones, it is difficult to give a quantitative description of the differences in clone sizes in the current study as we did not perform quantitative clonal analysis on a large enough number of clones to give statistically significant results. In addition to the trend in clone size reduction relative to time of infection, it was also apparent that, due to the sequential birth of retinal cell types, later injections resulted in gene transfer into a reduced number of cell types per clone, as seen previously and as predicted by birthdating studies (Young, 1985; Turner and Cepko, 1987; Turner et al., 1990).

Four different viral vectors were tested for expression in retinal cell types. Some promoters, including viral LTR promoters, can be silenced in certain cell types, and silencing can vary depending upon time of introduction into a tissue (Jaenisch, 1980; Gaiano et al., 1999). The four vectors were FUGW (Lois et al., 2002), LIA (Bao and Cepko, 1997), BAG (Price and Thurlow, 1988), and pQCXIX (Clontech). FUGW is a lentiviral vector with inactive LTR promoters. It expresses cytoplasmic GFP from an internal human ubiquitin C promoter. LIA and BAG are derived from the type C retrovirus Moloney murine leukemia virus (MMLV) and express human placental alkaline phosphatase and *β-galactosidase*, respectively, from slightly different MMLV LTRs. The vector pQCXIX is similarly derived from MMLV, but has inactive LTRs, and it expresses nuclear green fluorescent protein (GFP; pQC-H2BGFP-IX) from an internal CMV promoter (see the Experimental Procedures section). It, like LIA, has an internal ribosomal entry site (IRES) element allowing for expression of two genes from a bicistronic mRNA.

FUGW-infected retinas were difficult to identify due to low expression levels of GFP. While expression was seen in all expected cell types, independent of the injected stage, levels were low and only after antibody staining with an α-GFP antibody were the cell types detected. LIA (Fig. 2A–C) did not yield the expected cellular distribution within large clones after infection at E9.5–E10.5, but did give the expected results from infections at or after E12.5. Many clones from injections at E9.5–E10.5 lacked inner nuclear layer cells, although many photoreceptors were labeled (Fig. 2B), suggesting inactivation of the LTR in some cell types. BAG (Fig. 2D–I) and pQCXIX gave the expected result of large, complex clones with labeling of many cell types in each clone, after infection at all embryonic ages tested, with the exception that bipolar and possibly ganglion cells were weakly labeled by pQCXIX (Fig. 3A–C). Animals infected with pQCXIX were easily identified without dissection as the fluorescent signal from retinal clones could be visualized through the lens of the eye (Fig. 3D). After enucleation, examination of the eye revealed nuclear GFP not only in retinal cells, but also in the surrounding RPE and scleral tissue (Fig. 3E), which was confirmed by analysis of sections (data not shown). Promoter activity was followed up to 10 weeks postnatal with no noticeable decrease (Fig. 3F).

**Fig. 4:**
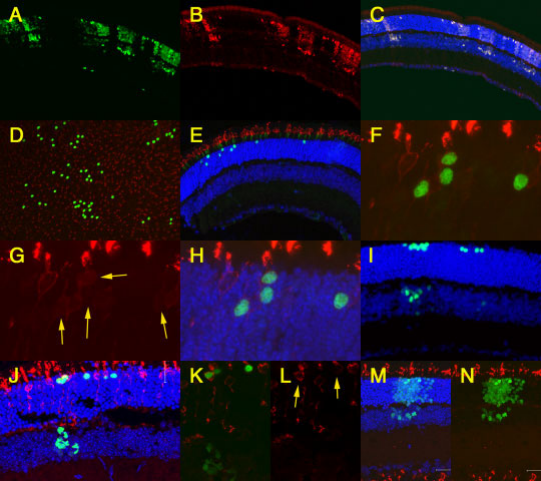
Multiple applications of embryonic day (E) 9.5 viral infections with pQCXIX. **A**–**C**: Postnatal day (P) 28 retina. pQCXIX promotes coexpression due to an internal IRES, with a nuclear green fluorescent protein (GFP, A) 5′ of the IRES and a membrane-Cherry (B) 3′ of the IRES (pQC-H2BGFP-I-mCherry). **C**: Overlay of A and B. D–H: Cell type-enriched expression in cones after removal of the CMV promoter and replacement with the cone arrestin promoter (pQmCAR-H2BGFP-IX). Red shows immunohistochemical signal for red/green opsin. **D**: An infected retina (P28) prepared as a flat mount showing individual cones with nuclear GFP. **E**: Low magnification of a cross-section at P28. Non-nuclear green signal shows cone segments labeled with PNA. **F**,**G**: High magnification of cross-section of retina at P13 showing expression of nuclear GFP (F) in cells that also express red/green cone opsin (G, arrows mark cells that were GFP positive in F). **H**: Overlay of F with 4′,6-diamidine-2-phenylidole-dihydrochloride (DAPI). I–L: Absence of rods presumably due to RNAi-mediated rod cell death in rd1 animals heterozygous for the rod-specific gene, PDE6b. Animals were infected at E9.5 with a virus carrying an shRNA for PDE6 (pQC-H2BGFP-IX-RNAiPDE6b). Red shows immunohistochemical signal for red/green opsin. **I**,**J**: Two different cross-sections at P35 showing only inner nuclear layer cells and presumably cones. **K**,**L**: Higher magnification of a single confocal section from retina in J showing inner nuclear layer cells and two cones (K) positive for red/green opsin (L, arrows). **M**,**N**: Section of rd1 animals heterozygous for the rod-specific gene PDE6b infected with a control virus expressing only nuclear GFP (pQC-H2BGFP-IX). In addition to the inner nuclear layer cells, many rods are positive for nuclear GFP. N: Same section as in M without DAPI.

To determine whether the biscistronic mRNA of pQCXIX was able to direct significant levels of coexpression, nuclear GFP and membrane-Cherry (Shaner et al., 2004) were cloned into the vector, with GFP 5′ of the IRES and cherry 3′ of the IRES (see the Experimental Procedures section). Both genes were easily visualized in the same cells, after infection at E9.5 (Fig. 4A–C). This finding suggests that coexpression of a gene of interest to investigate gene function, with a fluorescent reporter gene, should be successful. We further tested whether pQCXIX could be engineered to give cell type specificity and whether it could support RNAi-mediated loss-of-function. Viral vectors can be engineered to direct expression to a specific cell type by insertion of cell type-specific promoters into vectors with inactive LTRs, such as pQCXIX. To examine whether this could be achieved after embryonic infection, the CMV promoter of the pQCXIX vector was replaced with the cone arrestin promoter (Zhu et al., 2002; see the Experimental Procedures section). Strong expression in cones was seen after infection at E9.5–E10.5 (Fig. 4D–H). However, there was some weak to strong labeling of inner nuclear layer cells in some clones, as well as some rod labeling in some clones. The expression intensity of nuclear GFP in cell types other than cones varied among clones, with many having no expression except in cones, suggesting a positional effect of nearby genomic elements on the cone arrestin promoter.

The efficacy of RNAi-mediated loss-of-function in the pQCXIX vector was tested by using an shRNA to a rod-specific gene, phosphodiesterase beta (Pde6b), whose loss-of-function mutations in mice results in the death of rods (Bowes et al., 1990). The shRNA was driven from a U6 promoter that was inserted upstream of the CMV promoter pointing in the opposite direction (see the Experimental Procedures section). Infections of animals heterozygous for Pde6b (rd1 mice) at E9.5–E10.5 with pQCXIX encoding a shRNA to Pde6b resulted in clones with no rods (Fig. 4I–L). Because almost all control clones contain many rods (Fig. 4M,N), the absence of rods is presumably due to their death.

#### Gene transfer by electroporation

Electroporation provides for the delivery of plasmids to cells at the injection site. It has been successfully used for delivery to postnatal ocular structures in mice and rats, and embryonic eyes of chicks (Schulte et al., 1999; Matsuda and Cepko, 2004). We explored whether this method could be used to transduce embryonic murine ocular structures at different times. The uterine wall is very thick at E9.5–E10.5, preventing visualization of the embryo after removal of the ultrasound scan-head, which is necessary for proper placement of the electroporation electrodes. Therefore, electroporations were not performed before E12.5. After E12.5, the thickness of the uterine wall was reduced, and the embryo's size was larger, allowing for visualization and placement of the electrodes. More importantly, survival rates were very low after attempts to electroporate at E9.5–10.5.

At or after E12.5, electroporation into the eye was successful, and several aspects of this method were thus explored. Two differences between electroporation and viral infection are the number of targeted cells, the distribution of targeted cells, the stability of expression, and the types of constructs that can be used for gene transfer. Electroporation generally targets many more cells in a local area than viral infection, although this depends in large part on the viral titer and the skill of the investigator injecting the DNA. In addition, electroporated plasmids can be very large whereas viral constructs are typically more limited in size. Electroporation of large DNA plasmids such as phages, cosmids, or bacterial artificial chromosomes (BACs) should be applicable, as we have successfully used BACs for electroporation in newborn pups (Cherry and Cepko, unpublished observations). Additionally, coelectroporation of several plasmids allows simultaneous introduction of several genes into the same cells (Matsuda and Cepko, 2004,^2007^).

**Fig. 5:**
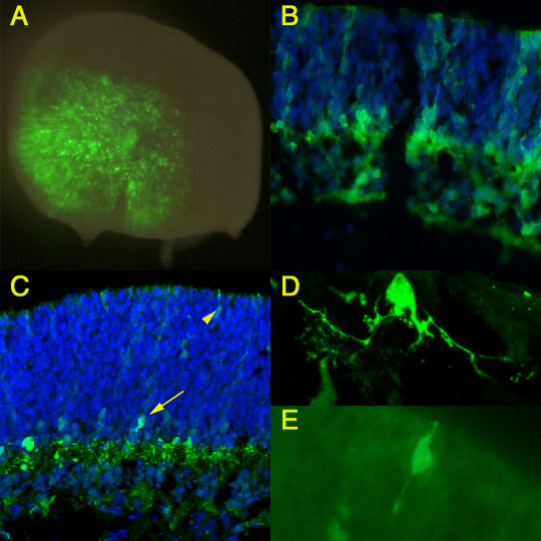
Gene transfer by electroporation. A–E: green fluorescent protein (GFP) fluorescence of embryos electroporated at embryonic day (E) 13 with pCAG-GFP. **A**: Dissected eye cup at E17.5 showing the electroporated area. B,C: Cross-sections through electroporated retinae. **B**: Section through retina shown in A at E17.5. **C**: By postnatal day (P) 2, most of the signal was found in amacrine cells (arrow) and developing amacrine processes in the inner plexiform layer, or in other early-born cell types, such as cones (arrowhead). **D**,**E**: High magnification at P2 showing an amacrine cell (D) and a cone (E).

The identification of retinae that were successfully targeted was enabled by electroporation of a plasmid encoding cytoplasmic GFP (Fig. 5A). This showed the typical arrangement of electroporated cells in which they were clustered in a targeted domain. Retinae that were electroporated at E13 were easily identified at E17 (Fig. 5A), but by postnatal day 2, the overall signal after dissection was very weak. By that time, the brightest signal was seen in the amacrine cells and their processes in the developing inner plexiform layer of the retina (Fig. 5C). This finding presumably was a reflection of the fact that plasmids do not integrate and are thus diluted at each cell division. Cells that become postmitotic shortly after electroporation inherit the nonintegrated plasmid, but do not dilute it further with additional cell divisions. Because amacrine cells are the most abundant of the cell types born shortly after the electroporation, they remain strongly labeled (Fig. 5D) after embryonic electroporation, as do some cone photoreceptors (Fig. 5E), which also have embryonic birthdays (Carter-Dawson and LaVail, 1979; Young, 1985).

## DISCUSSION

Ultrasound-guided in utero injections allow for studies of eye development that were not feasible previously. This includes spatiotemporal control of both gain- and loss-of-function. The choice of gene delivery method, by means of virus or electroporation, depends upon the application and should be chosen according to the age of delivery, number of cells to be targeted, stability of expression, size of construct(s), and whether or not multiple constructs need to be delivered. Viral infections at E9.5–E10.5 usually resulted in large clones composed of multiple columns of densely packed retinal cells, which should enable studies of retinal physiology. Similarly, studies of degeneration should be possible, because by adjusting the viral titer, clones covering up to 20% of the retinal surface area can be achieved. Animals infected with pQCXIX could be easily identified by GFP signal through the lens and thus can be selected for follow-up studies, for example, for physiology. In addition to gene transfer into the retina, these methods also yielded gene transfer of the RPE and sclera. Gene transfer into other anterior chamber structures such as the lens, the cornea, and the ciliary margin should also be possible, facilitating studies of glaucoma, cataract, and corneal development and diseases.

Gain-of-function by viral infection can be performed by overexpression of a gene in a broad or cell type-specific manner by use of a cell type-specific promoter that drives expression of the gene of interest. Cell type-specific overexpression can also be achieved by infecting a Cre-expressing mouse line with a virus where a flox-stop cassette precedes the gene of interest. Similarly, a virus with a cell type-specific promoter that precedes a flox-stop cassette in front of the gene of interest injected into a Cre-line with a different promoter will result in overexpression at the intersection of two different promoters. Gain-of-function by electroporation is straight forward as multiple plasmids can be electroporated at the same time. In addition, spatiotemporal control without the use of Cre-lines has been reported successfully for electroporations (Matsuda and Cepko, 2007). Analogous to gain-of-function, loss-of-function can be achieved by introducing a Cre or Flp by means of viral infection or electroporation into an animal engineered to delete a locus of interest. An alternative approach is to deliver RNAi, by means of a retroviral vector or electroporation.

Ultrasound-guided in utero injections during mouse eye development provides a more rapid screening process for novel gene function and promoter activity than previously afforded by the use of genetically modified mice. The ability to perform experiments, including the generation of loss-of-function clones similar to those that have advanced the understanding of *Drosophila* eye development, will enable scientists to dissect the cellular interactions that govern eye development in mouse. Screening for disease models generated by RNAi- or Cre-mediated loss-of-function, as well as rescue experiments in retinal degeneration mutants, should be straightforward. In addition, it is likely that these methods can be adapted to other species, thereby expanding the production of disease models to organisms where germline engineering is impossible or impractical. While this work was in progress, Garcia-Frigola et al., reported successful electroporation in utero into the embryonic mouse eye (Garcia-Frigola et al., 2007). However, they did not use ultrasound-guided injections and expression was confined mainly to amacrine and ganglion cells. Due to the lack of ultrasound-guided assistance, delivery of genes earlier than E13 is also not feasible by their method as the developing eye is too small to be targeted.

## EXPERIMENTAL PROCEDURES

### Animals

CD1, FVB/N, and C57Bl/6N were purchased from Charles River Laboratories, Inc. All procedures involving animals were in compliance with the ARVO Statement for the Use of Animals in Ophthalmic and Vision Research.

### Surgical Procedure and Viral Injections

Survival rates and target efficiencies are presented in Table 2. Survival rates for pregnant animals of FVB/N and C57Bl6 were 90% (18/20) and 87% (7/8), respectively. Surgeries were performed as previously described (Liu et al., 1998) with some minor modifications. Mice were anesthetized under constant Isofluorene flow according to the manufacturer's directions. Injections were performed at all stages directly through the uterine wall either into the diencephalon (E9.5–E10.5) or into the subretinal space (>E12.5) while the needle was monitored by ultrasound. The imaging system used (Vevo770) with a resolution of 30 μm, including surgical platform, microinjection apparatus, glass needles (Custom 0) with a 50-μm beveled tip for injections, scan-head holder, vaporizer, and accessories, were purchased from Visual Sonics (http://www.visualsonics.com). After the incision of the skin and peritoneal muscle, a sterile gauze with an incision was placed over the opened body cavity and wetted with sterile phosphate buffered saline (PBS). At E9.5–E10.5, one horn at a time was removed from the body cavity and placed on sterile gauze. At later stages (>E12.5), three to four embryos at a time were placed onto the gauze. Embryos or horn were covered directly with sterile ultrasound gel, imaged, and injected with either 0.6 μl of virus at E9.5–E10.5 into the diencephalon or 0.4 μl of virus at >E12.5 into the subretinal space. The needle was prefilled with mineral oil and front loaded before use, with sufficient virus to inject at least four embryos. After injection, the gel was removed with a squeeze bottle containing sterile PBS and embryos were placed back into the body cavity. The procedure was repeated with the second horn or with additional embryos of the same horn if only few embryos were removed instead of the entire horn. At the end of the injection procedure, the skin and peritoneal muscle were sutured individually. A single injection of 0.05 mg/kg body weight of buprenorphine was administered after the surgery and repeated for an additional 3 times in 12-hr intervals.

**Table 2:** Summary of Plasmids Used for Gene Transfer Either by Viral Infection or Electroporation^a^

Virus	Promoter	Reporter; localization	Injected at	Figure
LIA	LTR	Alkaline phosphatase; membrane	E9.5	2A, 2B
			E14.5	2C
BAG	LTR	β-galactosidase; cytoplasm	E9.5	2D, 2E, 2G, 2H
			E14.5	2F, 2I
FUGW	Ubiquitin C	GFP; cytoplasm	E9.5	not shown
			E13.5	
pQC-H2BGFP-IX	CMV	GFP; nuclear	E9.5	3B-3I
				4M, 4N
pQC-H2BGFP-I-mCherry	CMV	GFP; nuclear	E9.5	4A-4C
		Cherry; membrane		
pQmCAR-H2BGFP-IX	Cone arrestin	GFP; nuclear	E9.5	4D-4H
pQC-H2BGFP-IX-RNAiPDE6b	CMV	GFP; nuclear	E9.5	4I-4L
Electroporation plasmid				
pCAG-GFP	CAG	GFP; cytoplasm	E13	5A-5E

a Promoter, marker gene, time point of injection, and corresponding figures are indicated. GFP, green fluorescent protein; E, embryonic day.

### Electroporation

For electroporations (>E12.5), the gel was washed off (PBS squeeze bottle) from the embryo to be electroporated. The positive pole of the electrode was placed over the side of the eye and the negative on the other side of the head. Five pulses of 50 msec with 950-msec intervals were applied. Voltage was adjusted to the age of the embryo and may be further adjusted to different strains. Ideally, electroporation tests without injections allow determination of the best conditions for a particular strain and electroporation setting by assessing survival first. Recommended voltages are as follow: E12.5, 20–25 V; E13.5–E14.5, 25–30 V; E15.5 on, 35–40 V. DNA concentration ranged between 1 and 1.5 μg/μl with a volume of 0.4 μl injected into the subretinal space. The needle was usually front loaded as for viral injections but can also be back loaded if desired. After injection, the embryo was immediately electroporated before moving to the next one. The plasmid used for electroporation expresses a cytoplasmic GFP under the control of the CAG promoter (Matsuda and Cepko, 2004). Electroporation device ECM830 and tweezer-type electrodes (model 520, 7 mm diameter) were purchased from BTX, San Diego.

### Virus Work and Constructs

Viral preparations and concentration through centrifugation were performed as described previously (Cepko, 1989). Titers obtained with FUGW ranged between 5 × 10^6^ and 2 × 10^7^ CFU/ml. Titers obtained with LIA ranged from 5 × 10^5^ to 10^7^ CFU/ml. Titers obtained with BAG ranged between 10^6^ and 2 × 10^7^ CFU/ml. LIA and BAG require a histochemical reaction (Turner et al., 1990; Fields-Berry et al., 1992); therefore, all eyes of the litter have to be processed to determine the infected ones. Additionally, the strong staining of the alkaline phosphatase and *β-galacotsidase* reaction throughout the membrane and cytoplasm, respectively, can complicate interpretations of densely packed clonally related cells. Unless used in combination with antibodies, they are not suitable for fluorescent microscopy and double or triple labeling. The vector, pQCXIX has no marker gene but allows for insertion of two genes (at the position X). It, like LIA, has an IRES (here abbreviated I) element allowing for expression of two genes from a bicistronic mRNA. pQCXIX was purchased from Clontech (catalog no. 631515). A test vector was generated with a histone GFP fusion (H2BGFP) inserted into the first multiple cloning site (MCS) and a new extended second MCS. Titers with pQCXIX ranged from 5 × 10^6^ to 2 × 10^8^ CFU/ml. Viruses made from the construct that replaced the CMV promoter with the cone arrestin promoter (Zhu et al., 2002) cannot be titered by determining the amount of infectious particles, due to lack of expression in cell culture. To estimate the viral titer, the virus with the CMV promoter and the one with the cone arrestin promoter were made in parallel and the relative amount of viral genome was assessed by quantitative real-time polymerase chain reaction (PCR), which was then compared with the titer of infectious particles for the virus with the CMV promoter. The H2BGFP fusion construct was performed by PCR. *Not*I was added at the 5′ end and *Mnu*I at the 3′ end and the fusion was cloned into the first MCS of pQCXIX into *Not*I and *Eco*RI to generate pQC-H2BGFP-IX. The following restriction sites were added into the second MCS between *Mlu*I and *Xho*I: *Bsi*wI, *Sgr*AI, *Acc*III, *Mnu*I, *Pac*I (Supplementary Map/Sequence, which can be viewed at http://www.interscience.wiley.com/jpages/1058-8388/suppmat). The cone arrestin promoter (Zhu et al., 2002) was obtained by PCR from genomic DNA. An *Xba*I site and a *Not*I site were added at the 5′ end and at the 3′ end, respectively, and cloned into *Xba*I and *Not*I of pQC-H2BGFP-IX by replacing the CMV promoter to generate the pQmCAR-H2BGFP-IX (Supplementary Map/Sequence). The following targeting sequence was used for the PDE6b shRNA construct: 5′-GGGCCTGTGAAGATGGTTGGCA-3′and cloned into pBS/U6 (kindly provided by Dr. Yang Shi, Harvard Medical School, Boston, MA). The U6 promoter and the shRNA construct was excised as an *Xba*I cassette and cloned into *Xba*I of pQC-H2BGFP-IX. Orientation was verified to select for clones where the U6 promoter pointed in the opposite direction as the CMV promoter (Supplementary Map/Sequence). *Mlu*I and *Xho*I were added at the 5′ and at the 3′ end of membrane-Cherry (mCherry was kindly provided by Dr. Botond Roska, FMI, Basel Switzerland) by PCR. mCherry was cloned as *Mlu*I–*Xho*I fragment into *Mlu*I–*Xho*I of pQC-H2BGFP-IX to generate pQC-H2BGFP-I-mCherry (Supplementary Map/Sequence).

### Immunohistochemistry

#### Whole-mount

Retinae were dissected in PBS, fixed for 30 min in 4% paraformaldehyde/PBS, washed 3 × 10 min in PBS, washed for 1 hr in PBT (PBS, 0.3% Triton 100), blocked for 1 hr in PBTB (PBT, 5% BSA), incubated overnight at 4°C with primary antibody in PBTB, washed 3 × 20 min with PBTB, incubated 2 hr with secondary antibody in PBTB, washed 5 × 20 min in PBT. Sections, dissection, fixation, and washes were performed as for whole-mount retinae, then retinae were equilibrated in increasing sucrose concentrations (from 5% to 30% sucrose/PBS), mounted in OCT, frozen, sectioned, re-hydrated in PBS for 30 min, and then the same procedure was used again as for whole-mount starting with the 1 hr wash step in PBT. Primary antibody dilutions: rabbit α-red/green opsin (Chemicon), 1:300; Lectin PNA conjugate Alexa-488, 1:400 (Molecular Probes); rabbit α-GFP, 1:1,000 (Molecular Probes). Secondary antibody dilutions were F(ab′)_2_ fragments (Jackson ImmunoResearch) 1:500 on sections or whole-mount.
